# Maternal and paternal anxiety during pregnancy: Comparing the effects on behavioral problems in offspring

**DOI:** 10.1371/journal.pone.0275085

**Published:** 2022-10-03

**Authors:** Mona Bekkhus, Yunsung Lee, Sven Ove Samuelsen, Stella Tsotsi, Per Magnus

**Affiliations:** 1 Promenta Research Centre, Department of Psychology, University of Oslo, Oslo, Norway; 2 Centre for Fertility and Health, Norwegian Institute of Public Health, Oslo, Norway; 3 Department of Mathematics, University of Oslo, Oslo, Norway; 4 Department of Physical Health and Aging, Norwegian Institute of Public Health, Oslo, Norway; Texas A&M University College Station, UNITED STATES

## Abstract

Prenatal maternal anxiety has been associated with both short and long-term mental health problems in the child. The current study aims to examine the association between maternal and paternal prenatal anxiety and behaviour problems in the child at 1.5 and 5 years, using three different approaches; (1) adjusting for covariates, (2) using fathers’ anxiety during pregnancy as a negative control, and (3) using a sibling-comparison design, controlling for unmeasured family factors. We used data from the Norwegian Mother, Father and Child Cohort Study (MoBa) is used. MoBa is a cohort consisting of about 114 000 pregnancies (about 34000 siblings) recruited from 1999 to 2008. Self-reported measures on maternal anxiety were obtained twice in pregnancy and 6 months after birth, while paternal anxiety was reported prenatally at 17^th^ weeks of gestation. Maternal reports on child behaviour problems were obtained at 1.5 and 5 years of age. Results suggests that prenatal exposure to maternal anxiety was associated with behaviour problems at 1.5 years: adjusted beta (β) = 0.13 (CI = 0.12, 0.15), and at 5 years: β = 0.11 (CI = 0.09, 0.14). However, paternal anxiety was also associated with behaviour problems at 1.5 years: β = 0.03 (CI = 0.01–0.03) and at 5 years β = 0.03 (CI = 0.02, 0.03). These associations were attenuated in the sibling comparison analyses: β = *-*0.02 (CI = -0.02–0.05) at 1.5 years and β = -0.05 (CI = -0.10, 0.02) at 5 years. In conclusions, the sibling analyses are not consistent with a direct effect of prenatal maternal anxiety on child behaviour problems. It is more likely that genetic or shared family environment explain this association.

## Introduction

Exposure to maternal stress during pregnancy, such as anxiety, is a known risk factor for a wide range of developmental outcomes, and is associated with shortened gestation, restricted fetal growth, and increased emotional and behavioral difficulties in offspring [1–3]. The mechanism linking maternal exposures during pregnancy and offspring outcomes has been suggested to operate via the programming hypothesis [4]. This theory proposes that stress in the mother stimulates the release of the stress hormones corticotrophin-releasing hormone (CRH), adrenocorticotropic hormone (ACTH), and glucocorticoids. These hormones cross the placenta barrier and influence fetal-brain development [5, 6]. The programming hypothesis suggests that if such exposure occurs during any sensitive period of fetal development, it may lead to increased risk of disease later in life [7]. According to the fetal programming hypothesis if such exposure occurs during any sensitive period of fetal development, it may lead to increased risk of a disease later in life [8]. For example, in a recent meta-analysis, maternal anxiety was found to increase the risk of socioemotional problems, such as behavior problems by 50% [9]. MacKinnon et al. found an increased risk of both hyperactivity and conduct disorder after exposure to prenatal distress [10]. These negative consequences of maternal prenatal anxiety have been suggested to continue beyond childhood [3]. However, solving the methodological problems by randomizing pregnant mothers to the exposure is clearly not possible.

Despite this evidence, the link between prenatal exposure to maternal anxiety and child behavior problems is not clear [11, 12], and findings have been mixed. On the one hand, DiPietro et al. [13] found that moderate anxiety in pregnancy was associated with more optimal child development, while others reported no association between maternal distress and child behavior problems [12]. Thus, the association between prenatal maternal anxiety and child behavior problems may be due to residual confounding either prenatally or postnatally. Prenatal maternal anxiety may for example relate to behavior problems only indirectly through postnatal maternal anxiety [14]. On the other hand, the association between prenatal maternal anxiety and child behavior could be due to genetic confounding (e.g., shared family effects).With regards to shared genes, while in our cohort a partial overlap in the genetic variance between maternal depression and child behavior problems has been previously demonstrated [15], such links have not been extensively studied in the context of maternal anxiety. As addressing residual confounding by randomizing pregnant mothers to the exposure is clearly not possible. Our purpose is therefore to investigate whether maternal prenatal anxiety is related to behavior problems using a non-exposed control design. We apply three approaches to account for unmeasured and measured confounding.

First, we adjust for covariates, previously shown to be related to behavior problems in the child, by multiple linear-regression analyses. For example, some studies have found prenatal anxiety and depression to be associated with birth-related outcomes, e.g. low birth weight or preterm birth [1]. Others have also found birth outcomes, such as low birth weight, to be associated with behavior problems in the child [16, 17]. Studies also suggest that the effect of exposure to maternal prenatal anxiety may not occur in infancy but at a later age [3]. Thus, whether maternal anxiety influences fetal development, in turn causing behavior problems later in childhood, remains unclear. Therefore, in the first analyses, we first controlled for several known prenatal and postnatal confounders.

Second, we used paternal prenatal anxiety as a negative control [11]. Thus, if there is a direct effect of maternal anxiety in utero, prenatal maternal anxiety should have a stronger effect on child-behavior problems than prenatal paternal anxiety. For example, Ramchandani and colleagues [18] found depression in fathers during the prenatal period to be associated with behavior problems in three-year-olds. They examined the association between prenatal and postnatal paternal depression for behavior problems. They did not, however, compare maternal prenatal depression with paternal prenatal depression, or examine anxiety. Van Batenburg-Eddes and colleagues [17] examined both maternal and paternal anxiety and depression in two large cohort studies and found similar effects of maternal or paternal anxiety/depression on child-inattention symptoms. Thus, their findings suggest that although there was a direct effect of prenatal maternal depression and anxiety, most of the observed effects could be explained by residual confounding (i.e., either paternal symptoms or postnatal effects of parental symptoms). However, they did not examine behavior problems.

A third approach to account for confounding factors is to apply a sibling-comparison design. This approach may provide an effective control for familial factors that are constant (maternal genes and family context). Two large-scale studies that compared siblings differentially exposed prenatally to depression [19] or adverse life-events [20] did not find any association to offspring behavior. Similar findings emerged when the association between maternal prenatal anxiety and child emotional difficulties at 6 and 36 months was examined using a sibling design [21]. These studies found that the association was confounded by shared family effects. However, as maternal prenatal anxiety may differentially relate to child internalizing versus externalizing versus neurodevelopmental outcomes [18, 22], the biological pathway from prenatal exposure to maternal anxiety on child behaviour may differ depending on the specific outcome. Based on these previous studies, maternal prenatal anxiety seems to have a stronger influence on the development of externalizing difficulties (i.e. behaviour problems) compared to internalizing ones (i.e. emotional difficulties) [18, 22]. Studies also suggest that the effect of exposure to maternal prenatal anxiety may not occur in infancy but at a later age [23].

Building upon this earlier evidence, in the present study we examine whether the association between maternal prenatal anxiety and child behaviour problems in early childhood can be attributed to measured (known covariates, paternal prenatal anxiety) and unmeasured (shared genetic variance and environment) confounding. We aim to examine the association between maternal prenatal anxiety and behavior problems, using three different approaches: (i) adjusting for covariates and postnatal anxiety as a negative control, (ii) using fathers’ anxiety during pregnancy as a negative control, and (iii) using a sibling-comparison design, controlling for unmeasured family factors. We aim to examine effects both in infancy and later in childhood by using behavior problems at both 1.5 and 5 years as the outcome.

## Materials and methods

This study is a subproject of the Norwegian Mother, Father, and Child Cohort Study (MoBa) conducted by the Norwegian Institute of Public Health [24]. MoBa is a cohort comprising 114,247 pregnancies recruited from 1999 to 2008 with a participation rate of 40.6% [25]. All but two of the 52 hospitals across Norway agreed to participate in the recruitment for the study. Women were invited to participate when they attended the routine ultrasound examination that is offered to all pregnant women at 17–18 weeks of gestation (www.fhi.no/moba-en). The maternal-questionnaire response rates at the 17^th^ and 30^th^ weeks of gestation, and at 1.5 and 5 years after birth, were 95.1%, 91.4%, 87.0%, and 54.0%, respectively [25]. Written informed consent was obtained from all participating women. The Regional Committee for Medical and Health Research Ethics approved the study.

Within MoBa, 59,292 mothers of 68,602 children completed a series of questionnaires for each unique pregnancy, at the 17^th^ and 30^th^ weeks of gestation, and at 6 months and 1.5years after birth. The questionnaires included maternal anxiety level (17^th^ and 30^th^ weeks of gestation and 6 months after birth), paternal anxiety level (17^th^ week of gestation), and infant-behavior problems (1.5 and 5 years), along with other maternal characteristics such as age, education, marital status, smoking and drinking habits, and partner harmony. MoBa was also linked to the Medical Birth Registry of Norway (MBRN), which contains detailed medical information about newborn children (e.g., sex, birth weight, and gestational age) as well as their mothers (e.g., parity and birth complications).

**Fig 1:**
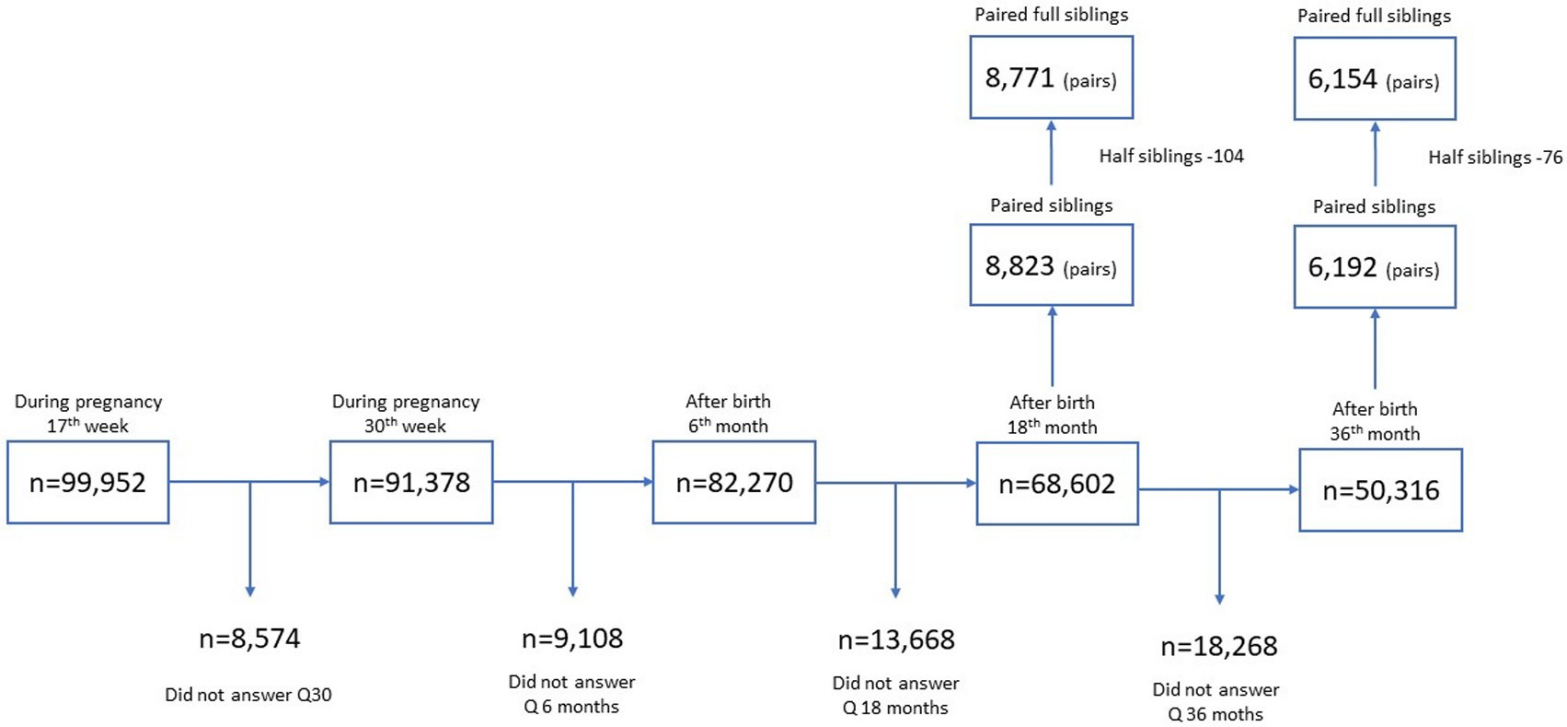
Flow chart of study sample. Note: The half siblings refer to the siblings with the same mother but different fathers.

In MoBa, there were 8,823 pairs of siblings at 1.5 years, and 3,428 of siblings at 5 years after birth with complete information. The mean age-difference between siblings was 2.7 years. The numbers dropped slightly after excluding half-siblings who had the same mother but different fathers (Fig 1). We used version 8 of the quality-assured data files; an overview of the questionnaires and items is available at https://www.fhi.no/en/studies/moba/for-forskere-artikler/questionnaires-from-moba/.

### Measures

#### Maternal and paternal anxiety

Mothers reported on symptoms of anxiety using six items from the validated short version of the Hopkins Symptom Checklist, two from SCL-5 and four from SCL-8 [26]. Items from the SCL are scored on a Likert scale ranging from 1 (not at all bothered) to 4 (very much bothered) and have been validated with a correlation of 0.92 with the SCL-25 [26]. Maternal anxiety was assessed twice during pregnancy (at the 17^th^ and 30^th^ gestational weeks) and once after birth (when the child was six months old). The mean scores for the 17^th^ and the 30^th^ gestational weeks were calculated separately using the items of constantly “frightened or anxious,” “nervous, inner turmoil,” “feel tense or stressed,” and “sudden fear without reason.” The maternal prenatal anxiety score was defined as the mean of the anxiety score at the 17^th^ and 30^th^ weeks. Paternal anxiety was also assessed once at the 17^th^ week of gestation using four items from the SCL-8.

#### Child-behavior problems

Child-behavior problems were measured by items from the Child Behavior Checklist (CBCL/TRF) [27]. A team of clinical and developmental psychologists selected the items from the CBCL used in MoBa. The selected items were based on theoretical and empirical representativeness and are representative with a correlation of 0.92 with the full scale [28]. Mothers responded to 8 items at 1.5 years and 10 items at 5 years on a three-point Likert scale, from not true (“0”) to sometimes true (“1”) to often true (“2”), and mean values were computed for each time point. Cronbach’s alpha was 0.57 and 0.74 for 1.5 years and 5 years, respectively.

#### Potential confounders

**Fig 2:**
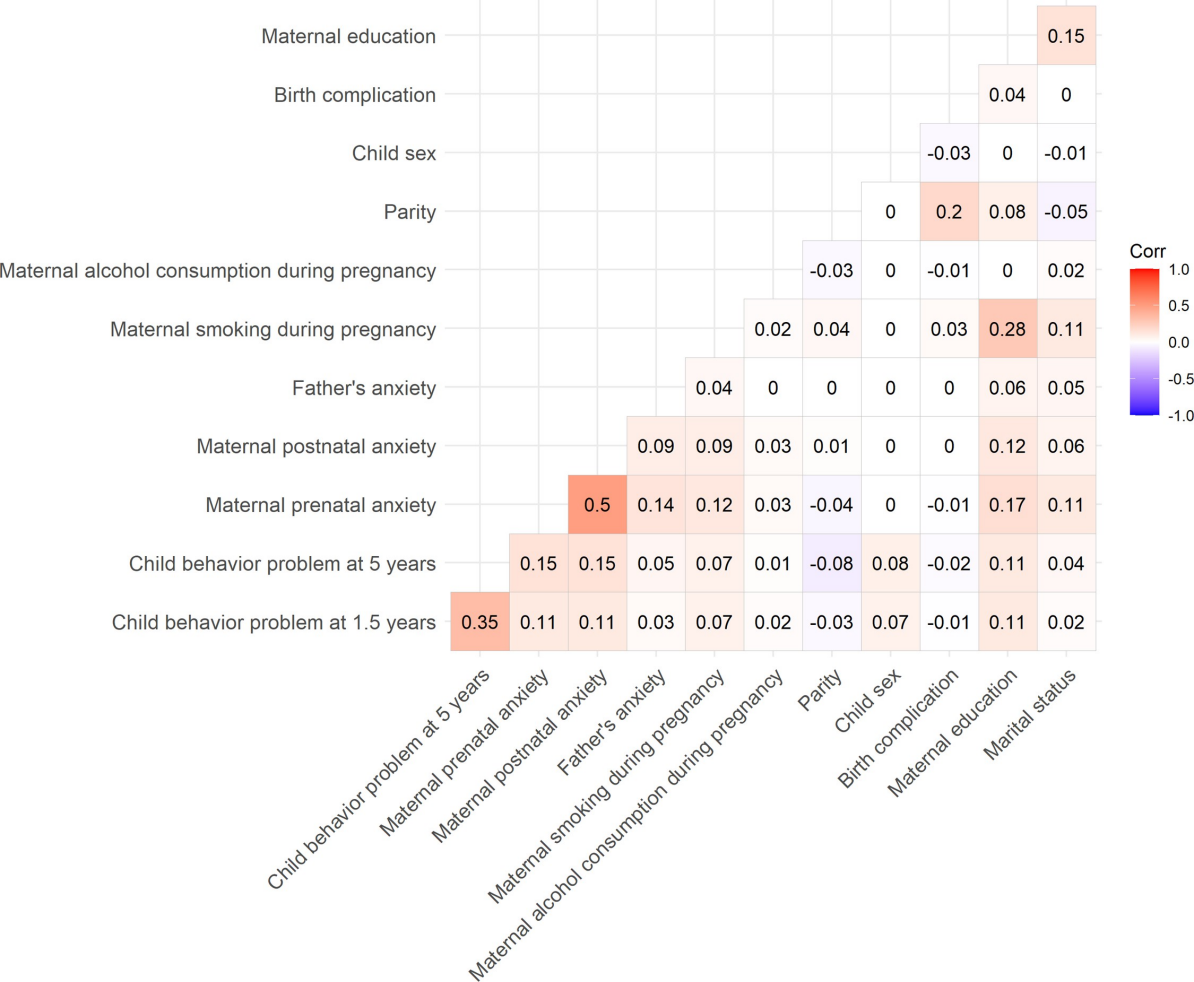
Pearson correlations between study variables.

The following variables were considered to be potential confounders: alcohol consumption during pregnancy (coded as never “0” and more than once a month “1”), smoking in pregnancy (coded as never “0,” sometimes “1,” and daily “2”), 10 items regarding partner harmony from the Marital Satisfaction Scale (MSS) with response options ranging from 1 to 6 (the higher, the worse), marital status (married/living together “0” and single “1”), and low maternal education (coded as higher university degree +4 years “0,” college/university 3 years “1,” college 1–2 years “2,” and secondary school “3”). We also controlled for the following variables extracted from the MBRN: parity (coded as 0, 1, 2, 3, and 4+), birth complications (coded as yes “1” or no “0”), and child sex (girl “0,” boy “1”). The Pearson correlations between the variables can be found in Fig 2.

### Statistical analyses

In the full cohort, the associations of maternal anxiety with child-behavior problems at 1.5 and 5 years were estimated using two different regressions: 1) ordinary linear regression of the child-behavior problem score on the maternal-anxiety score and 2) logistic regression of the dichotomized child-behavior problem score on the dichotomized maternal-anxiety score. The two regression models were adjusted for maternal smoking, alcohol consumption, parity, parental education, marital status, birth complication, and the interaction term between maternal prenatal and postnatal anxiety. When the outcome variable was the child-behavior problem measured at 5 years, the regressions were further adjusted for the child-behavior problem at 1.5 years.

Adjusted model:

$$Y_{child-behavior\;at\;1.5yrs}=\beta_{0}+\beta_{1}X_{mat-anx-pre}+\beta_{2}X_{mat-anx-post}+\beta_{3}\left(X_{mat-anx-pre}X_{mat-anx-post}\right)+\beta_{4}X_{mat-smk}+\beta_{5}X_{mat-alc}+\ldots +\epsilon$$

$$Y_{child-behavior\;at\;5yrs}=\beta_{0}+\beta_{1}X_{mat-anx-pre}+\beta_{2}X_{mat-anx-post}+\beta_{3}\left(X_{mat-anx-pre}X_{mat-anx-post}\right)+\beta_{4}Y_{child-behavior\;at\;1.5yrs}+\beta_{5}X_{mat-smk}+\beta_{6}X_{mat-alc}+\ldots +\epsilon$$

Additionally, we focused on the association between the maternal/paternal anxiety score at the 17^th^ week and the child-behavior problem. The child-behavior problem score was regressed on the maternal/paternal anxiety score at the 17^th^ week with adjustment for the paternal/maternal anxiety score at the 17^th^ week and the same set of adjusting variables used in the full cohort.

Mutually adjusted model:

$$Y_{child-behavior\;at\;1.5yrs}=\beta_{0}+\beta_{1}X_{mat-anx\_w17}+\beta_{2}X_{pat-anx-w17}+\epsilon$$

$$Y_{child-behavior\;at\;5yrs}=\beta_{0}+\beta_{1}X_{mat-anx-w17}+\beta_{2}X_{pat-anx-w17}+\epsilon$$

Fully adjusted model:

$$Y_{child-behavior\;at\;1.5yrs}=\beta_{0}+\beta_{1}X_{mat-anx-w17}+\beta_{2}X_{pat-anx-w17}+\beta_{3}X_{mat-anx-6m}+\beta_{4}X_{mat-smk}+\beta_{5}X_{mat-alc}+\ldots +\epsilon$$

$$Y_{child-behavior\;at\;5yrs}=\beta_{0}+\beta_{1}X_{mat-anx-w17}+\beta_{2}X_{pat-anx-w17}+\beta_{3}X_{mat-anx-6m}+\beta_{4}X_{child-behavior\;at\;1.5yrs}+\beta_{5}X_{mat-smk}+\beta_{6}X_{mat-alc}+\ldots +\epsilon$$

In the sibling design, the association between maternal anxiety and the child-behavior problem was estimated with an adjustment for the family-shared effect. We used two types of regression: 1) regression of the inter-sibling difference in the child-behavior score on the inter-sibling difference in the maternal anxiety score and 2) conditional-logistic regression of the dichotomized child-behavior problem score on the dichotomized maternal-anxiety score (fixed effect) and family identification (random effect). The same set of adjusting variables mentioned above was used.

Adjusted model:

- Contrast regression
$$\left(Y_{child-behavior\;at\;1.5\;yrs,\;sib1}-Y_{child-behavior\;at\;1.5yrs,\;sib2}\right)=\beta_{1}\left(X_{mat-anx-pre,\;sib1}-X_{mat-anx-pre,\;sib2}\right)+\beta_{2}\left(X_{mat-anx-post,\;sib1}-X_{mat-anx-post,\;sib2}\right)+\beta_{3}\left(X_{mat-anx-pre,\;sib1}-X_{mat-anx-pre,\;sib2}\right)\left(X_{mat-anx-post,\;sib1}-X_{mat-anx-post,\;sib2}\right)+\beta_{4}\left(X_{mat-smk,\;sib1}-X_{mat-smk,\;sib2}\right)+\ldots +\epsilon$$
- Conditional logistic regression:
$$logit\left(P\left(Y_{child-behavior\;at\;1.5yrs}=1\right)\right)=\beta_{1}X_{mat-anx-pre}+\beta_{2}X_{mat-anx-post}+\beta_{3}X_{mat-anx-pre}X_{mat-anx-post}+\beta_{4}X_{mat-smk}+\;\beta_{5}X_{mat-alc}+\ldots +\alpha_{family-ID}$$

### Missing data

Information about the proportion of missing data is given in Tables 1 and 2. Missing data in MoBa have previously been examined by Nilsen et al. [29].

**Table 1:** Characteristics of full cohort.

		Behavior Problems at 1.5 years			Behavior Problems at 5 years		
		Counts n = 73,757	Mean *μ* = 0.49	(SD) (s = 0.28)	Counts n = 40,163	Mean *μ* = 0.35	(SD) (s = 0.28)
**Mother’s prenatal anxiety**	1–2, Light	70,072	0.49	(0.28)	38,293	0.35	(0.28)
**(score)**	2–3	2,989	0.57	(0.31)	1,490	0.46	(0.33)
	3–4, Severe	308	0.58	(0.33)	132	0.51	(0.37)
	NA	388	0.54	(0.34)	248	0.39	(0.31)
**Maternal postnatal**	1–2, Light	67,552	0.48	(0.28)	37,053	0.35	(0.28)
**Anxiety**	2–3	2,761	0.57	(0.31)	1,260	0.47	(0.34)
**(score)**	3–4, Severe	361	0.59	(0.32)	167	0.50	(0.36)
	NA	3,083	0.52	(0.30)	1,683	0.38	(0.31)
**Father’s anxiety**	1–2, Light	53,910	0.49	(0.28)	32,526	0.35	(0.28)
**(score)**	2–3	1,216	0.51	(0.29)	748	0.40	(0.30)
	3–4, Severe	123	0.53	(0.32)	61	0.43	(0.31)
	NA	18,508	0.49	(0.29)	6,828	0.36	(0.29)
**Smoking**	None	64,344	0.48	(0.28)	36,058	0.35	(0.28)
**during pregnancy**	Sometimes	2,928	0.54	(0.29)	1,259	0.41	(0.30)
	Daily	2,543	0.56	(0.31)	911	0.46	(0.31)
	NA	3,942	0.49	(0.30)	1,935	0.36	(0.29)
**Alcohol consumption**	Never	60,103	0.49	(0.28)	33,809	0.35	(0.28)
**during pregnancy**	1+/month	435	0.54	(0.28)	265	0.40	(0.30)
	NA	13,219	0.49	(0.30)	6,089	0.35	(0.29)
**Parity**	0	34,199	0.49	(0.28)	19,057	0.38	(0.29)
	1	25,626	0.50	(0.29)	13,984	0.35	(0.28)
	2	10,938	0.46	(0.28)	5,645	0.31	(0.27)
	3	2,298	0.46	(0.29)	1,138	0.31	(0.28)
	4+	696	0.46	(0.29)	339	0.30	(0.26)
**Child sex**	Boy	37,709	0.51	(0.29)	20,491	0.38	(0.30)
	Girl	36,047	0.47	(0.28)	19,672	0.33	(0.27)
	NA	1	1.40	-	0	-	-
**Birth complication**	No	53,581	0.49	(0.28)	29,827	0.36	(0.29)
	Yes	20,176	0.48	(0.28)	10,336	0.34	(0.28)
**Education**	University 4+	46,673	0.47	(0.27)	27,704	0.33	(0.27)
	College/University 3y	18,494	0.53	(0.29)	8,520	0.40	(0.30)
	College 1-2y	3,013	0.56	(0.30)	1,163	0.44	(0.32)
	Secondary school	1,393	0.58	(0.31)	514	0.46	(0.32)
	NA	4,184	0.50	(0.29)	2,262	0.37	(0.30)
**Marital status**	Married/Partner	71,097	0.49	(0.28)	38,785	0.35	(0.28)
	Single	2,660	0.52	(0.30)	1,378	0.41	(0.31)

**Table 2:** Characteristics of sibling sub-sample.

		Behavior Problems at 1.5years			Behavior Problems at 5 years		
		Counts n = 18,767	Mean *μ* = 0.48	(SD) (s = 0.28)	Counts n = 10,922	Mean *μ* = 0.33	(SD) (s = 0.27)
**Mother’s prenatal anxiety**	1–2, Light	18,143	0.47	(0.28)	10,596	0.32	(0.27)
**(score)**	2–3	495	0.57	(0.32)	247	0.43	(0.34)
	3–4, Severe	56	0.48	(0.29)	24	0.40	(0.29)
	NA	73	0.56	(0.29)	55	0.35	(0.25)
**Maternal postnatal**	1–2, Light	17,592	0.47	(0.28)	10,251	0.32	(0.27)
**Anxiety**	2–3	487	0.56	(0.31)	250	0.43	(0.35)
**(score)**	3–4, Severe	68	0.57	(0.33)	37	0.44	(0.39)
	NA	620	0.48	(0.28)	384	0.33	(0.28)
**Father’s anxiety**	1–2, Light	15,369	0.47	(0.28)	9,381	0.33	(0.27)
**(score)**	2–3	274	0.48	(0.29)	172	0.35	(0.27)
	3–4, Severe	25	0.52	(0.33)	12	0.37	(0.37)
	NA	3,099	0.48	(0.29)	1,357	0.33	(0.27)
**Smoking**	None	17,075	0.47	(0.28)	10,139	0.32	(0.27)
**during pregnancy**	Sometimes	474	0.54	(0.30)	190	0.39	(0.32)
	Daily	382	0.56	(0.30)	147	0.39	(0.31)
	NA	836	0.48	(0.28)	446	0.34	(0.27)
**Alcohol consumption**	Never	15,854	0.47	(0.28)	9,411	0.33	(0.27)
**during pregnancy**	1+/month	73	0.51	(0.29)	40	0.41	(0.29)
	NA	2,840	0.48	(0.29)	1,471	0.32	(0.28)
**Parity**	0	8,071	0.48	(0.27)	3,761	0.34	(0.27)
	1	7,834	0.48	(0.28)	5,238	0.33	(0.28)
	2	2,257	0.45	(0.27)	1,515	0.30	(0.26)
	3	458	0.45	(0.30)	309	0.29	(0.27)
	4+	147	0.43	(0.29)	99	0.30	(0.24)
**Child sex**	Boy	9,657	0.49	(0.28)	5,637	0.35	(0.28)
	Girl	9,110	0.46	(0.27)	5,285	0.31	(0.26)
	NA	0	-	-	0	-	-
**Birth complication**	No	13,464	0.48	(0.28)	7,692	0.33	(0.27)
	Yes	5,303	0.47	(0.28)	3,230	0.32	(0.27)
**Education**	University 4+	13,359	0.46	(0.27)	8,300	0.31	(0.27)
	College/University 3y	3,848	0.51	(0.28)	1,873	0.36	(0.29)
	College 1-2y	474	0.56	(0.30)	191	0.40	(0.31)
	Secondary school	184	0.57	(0.31)	66	0.56	(0.37)
	NA	902	0.50	(0.28)	492	0.35	(0.27)
**Marital status**	Married/Partner	18,433	0.48	(0.28)	10,761	0.33	(0.27)
	Single	334	0.46	(0.28)	161	0.33	(0.30)

## Results

### Descriptive

Table 1 presents descriptive statistics for the full cohort. The mean score of child-behavior problems at 1.5 years increased with the severity of maternal prenatal and postnatal anxiety and paternal anxiety. The mean score of child-behavior problems at 1.5 years increased from 0.49 to 0.58 as maternal prenatal anxiety shifted from light to severe. Similarly, the mean score of child-behavior problems at 5 years increased from 0.35 to 0.51 with the severity of anxiety for the mothers measured postnatally (six months after birth). The mean score of child behavior at both 1.5 and 5 years also increased with the severity of paternal anxiety, but the inclination was not as large as that with maternal anxiety.

Among the 18,767 siblings (Table 2) with complete data on child-behavior problems at 1.5 years, the mean score of child-behavior problems increased and decreased (0.47 → 0.57 → 0.48) as maternal prenatal anxiety escalated from light to moderate to severe. We observed a similar pattern in the mean score of child-behavior problems at 5 years. However, the mean score of child-behavior problems at both 1.5 and 5 years increased as maternal postnatal (or paternal) anxiety became severe.

### Prenatal and postnatal maternal anxiety

We found a moderately positive association between prenatal maternal anxiety and child-behavior problems at both 1.5 years (*β* = 0.14, 95% confidence interval [CI] 0.13, 0.16) and 5 years (*β* = 0.19, 95% CI 0.16, 0.21) (see Table 3). These associations remained significant after adjusting for potential confounders (maternal smoking, alcohol intake, parental education, parity, marital status, and birth complication). We also found moderate associations between postnatal maternal anxiety and child-behavior problems at both 1.5 years (*β* = 0.15, 95% CI 0.13, 0.17) and at 5 years (*β* = 2.0, 95% CI 0.17, 0.22). A similar pattern was observed when logistic regressions were applied.

**Table 3:** The effect of maternal anxiety on child behavior problems at 1.5 and 5 years (full cohort).

	Behavior Problems at 1.5 years		Behavior Problems at 5 years	
**Multiple linear regression**	Crude beta (95% CI)	Adjusted beta (95% CI)^1^	Crude beta (95% CI)	Adjusted beta (95% CI)^2^
Sample size (n)	n = 70,522	n = 52,175	n = 38,067	n = 27,224
No maternal anxiety	(reference)	(reference)	(reference)	(reference)
Prenatal anxiety only	0.14 (0.13, 0.16)	0.13 (0.12, 0.15)	0.19 (0.16, 0.21)	0.11 (0.09, 0.14)
Postnatal anxiety only	0.15 (0.13, 0.17)	0.15 (0.13, 0.173)	2.0 (0.17, 0.22)	0.13 (0.10, 0.16)
**Logistic regression**	Crude OR (95% CI)	Adjusted OR (95% CI)^1^	Crude OR (95% CI)	Adjusted OR (95% CI)^2^
Sample size (n)	n = 70,522	n = 52,175	n = 38,067	n = 27,224
No maternal anxiety	(reference)	(reference)	(reference)	(reference)
Prenatal anxiety only	1.5 (1.4, 1.6)	1.37 (1.25, 1.51)	1.87 (1.70, 2.07)	1.48 (1.30, 1.68)
Postnatal anxiety only	1.6 (1.5, 1.8)	1.60 (1.42, 1.79)	2.12 (1.88, 2.40)	1.68 (1.44, 1.97)

^1^ Adjusted for maternal smoking, alcohol, parental education, parity, marital status, birth complication, and the interaction term between maternal prenatal and postnatal anxiety.

^2^ Also adjusted for child behavior problems at 18 months.

^3^ All estimates are significant = *p<0*.*05*

### Fathers as a negative control

We compared the effects of maternal anxiety and paternal anxiety at the 17^th^ week on child-behavior problems at 1.5 and 5 years (Table 4) because paternal anxiety was assessed at week 17 only. The effect size of paternal anxiety on child-behavior problems was smaller than that of maternal anxiety. The effect size of maternal anxiety on child-behavior problems at 1.5 years (0.06, 95% CI 0.06, 0.07) was twice the effect size of paternal anxiety (0.03, 95% CI: 0.02, 0.04). These maternal and paternal effects were reduced after adjustment for potential confounders, but remained significant.

**Table 4:** The effect of prenatal anxiety on child behavior problems at 1.5 and 5 years.

	Behavior Problems at 1.5years			Behavior Problems at 5 years		
	Crude beta (95% CI)	Mutually adjusted^1^ beta (95% CI)	Fully adjusted^2^ beta (95% CI)	Crude beta (95% CI)	Mutually adjusted^1^ beta (95% CI)	Fully adjusted^3^ beta (95% CI)
**Maternal anxiety at 17th week**	0.06 (0.06, 0.07)	0.06 (0.06, 0.07)	0.03 (0.02, 0.03)	0.09 (0.08, 0.09)	0.08 (0.07, 0.09)	0.03 (0.02, 0.04)
**Paternal anxiety at 17th week**	0.03 (0.02, 0.04)	0.02 (0.02, 0.03)	0.02 (0.01, 0.03)	0.06 (0.05, 0.07)	0.05 (0.03, 0.06)	0.03 (0.02, 0.05)

^1^ Adjusted for either maternal or paternal anxiety at 17th week.

^2^ Adjusted for maternal anxiety 6 months, smoking, alcohol, parental education, parity, marital status and birth complication.

^3^ Adjusted for maternal anxiety 6 months, child behavior problems at 1.5 years, smoking, alcohol, parental education, parity, marital status and birth complication.

All estimates are significant at *p<* .*05*

### Sibling comparisons

The associations between maternal anxiety and child-behavior problems were adjusted for family-shared effects in the sibling design (Table 5). The moderate associations observed in the full cohort (Table 3) almost vanished in the sibling design. For example, the adjusted effect of prenatal anxiety on child behavior problems at 1.5 years was weak 0.02 (CI 95% -0.01, 0.05). The adjusted effect of prenatal anxiety on child-behavior problems at 5 years also appeared to be weak 0.02 (CI 95% -0.03, 0.05). Maternal prenatal and postnatal anxiety did not affect child-behavior problems after adjustment in this sibling design. We found consistent results from conditional logistic regressions.

**Table 5:** The effect of maternal anxiety on child behavior problems at 1.5 and 5 year (sibling design).

	Child behavior problems at 1.5 years		Child behavior problems at 5 years	
**Contrast regression**	Crude beta (95% CI)	Adjusted beta (95% CI)^1^	Crude beta (95% CI)	Adjusted beta (95% CI)^2^
Sample size (n)	(n = 7,378 pairs)	(n = 4,837 pairs)	(n = 3,237 pairs)	(n = 2,092 pairs)
No maternal anxiety	(reference)	(reference)	(reference)	(reference)
Prenatal anxiety only	-0.00 (-0.02, 0.03)	0.01 (-0.02, 0.05)	-0.02 (-0.06, 0.02)	-0.05 (-0.10, 0.01)
Postnatal anxiety only	0.01 (-0.01, 0.04)	0.02 (-0.01, 0.05)	0.01 (-0.03, 0.05)	0.02 (-0.03, 0.07)
**Conditional logistic regression**	Crude OR (95% CI)	Adjusted OR (95% CI)^1^	Crude OR (95% CI)	Adjusted OR (95% CI)^2^
Sample size (n)	(n = 18,122)	(n = 14,045)	(n = 10,526)	(n = 7,910)
No maternal anxiety	(reference)	(reference)	(reference)	(reference)
Prenatal anxiety only	1.06 (0.75, 1.50)	1.17 (0.74, 1.85)	1.16 (0.68, 1.97)	1.09 (0.52, 2.29)
Postnatal anxiety only	1.32 (0.88, 1.98)	1.07 (0.63, 1.80)	1.67 (0.90, 3.11)	1.36 (0.58, 3.19)

^1^ Adjusted for maternal anxiety 6 months, smoking, alcohol, parental education, parity, marital status, birth complication, and the interaction term between maternal prenatal and postnatal anxiety.

^2^ Also adjusted for child behavior problems at 1.5 years.

^3^ All estimates at not significant (*p* > .05)

## Discussion

In this study, we applied three different approaches to control for unmeasured and measured confounding when examining the link between maternal anxiety during pregnancy and child-behavior problems. We found that behavior problems were higher when children had been exposed to maternal anxiety during pregnancy. Mothers’ postnatal anxiety measured at six months was also associated with increased behavior problems at 1.5 and 5 years of age. Although the associations were somewhat weaker, they remained after adjusting for a number of potential confounders. The effect sizes are equivalent to those found in previous studies [9].

The second approach was to apply the father’s anxiety measured during pregnancy as a negative control. Paternal anxiety operates as a negative control based on the assumption that paternal anxiety during pregnancy does not have any direct effect on the intrauterine environment coupled with the assumption that confounds are equally associated with both paternal and maternal anxiety [11]. We found that paternal prenatal anxiety was associated with child behavior. This suggests that the association between parental anxiety and child behavior could be confounded by shared environmental or genetic effects. However, this association was weaker than for maternal anxiety. This finding is in line with previous studies using paternal anxiety as a negative control [30] and points to the possibility of a direct biological effect of maternal anxiety through the intrauterine environment on child behavior [11].

To account for shared family effects, we applied a sibling-comparison design. In the sibling-comparison analyses, no associations were found for maternal anxiety measured either prenatally or at six months after birth. The implication is that maternal genetic and family-environmental factors that are shared by siblings may explain the consistent associations found in epidemiological studies examining the fetal-programming hypothesis. This finding is in line with previous studies examining relationships between depression during pregnancy and offspring mental health [16, 19]. However, no previous studies have examined paternal anxiety as a negative control, the timing of exposure, and maternal anxiety and associations with short- and long-term child behavior.

### Strengths and limitations

A major strength of this study was applying a sibling-comparison design. Sibling designs can reveal spurious effects of the explanatory variables, since many of these factors are similar through successive pregnancies and thus exposed to each sibling. While non-twin sibling designs cannot completely control for genetic effects, they do offer a relatively effective control for shared genetic influences as well as non-measured shared family confounds. However, an advantage of the non-twin sibling design over the MZ-twin design is that it provides an opportunity to examine different environmental exposures in utero.

Another example is extrapolating from animal models to complex human traits. Whether the stress response in rodents and humans shares similar mechanisms is subject to debate. This is particularly so in light of findings that imply the possibility that effects of stress during pregnancy in humans may be due to confounding factors not taken into consideration in animal research. Because we used data from a large population-based dataset, we were able to control for a number of covariates, such as birth complications, smoking and alcohol consumption during pregnancy, and maternal age and education.

One of the limitations of our study is the potential lack of representation due to the selection bias of MoBa participants. However, a study examining the effect of recruitment bias using data from the MoBa study showed that even though prevalence estimates of exposures and outcomes were not always equal to that of the background population, estimates of associations were not biased [29]. The women participating with more than one pregnancy in the cohort might also represent an element of selection bias. As has been suggested by Sjölander et al., [30] there is a possibility that the first pregnancy influences the second. Additionally, since we used self-reports from the mothers, the associations may be biased by common-method variance. For instance, highly anxious mothers may be poor judges of their children’s difficult behavior compared to mothers experiencing less anxiety. However, since we examine the outcomes of young children, mothers were deemed to be the individuals with the most knowledge about their child’s behavior, outweighing the possibility of bias through halo effects. Using the same informant–the mother–is often unavoidable in large cohort studies. Furthermore, as the behaviors measured were mostly observable child behaviors, they seemed less liable to bias than other child traits might be.

A third limitation is that we used short scales with few items to measure anxiety when broader measures of anxiety and clinical diagnostic interviews would have been optimal. However, in large-scale studies with over 100,000 pregnancies, clinical interviews would be extremely time-consuming and costly. Furthermore, these items were found to correlate highly with the longer version of the shortened scale [28].

In sum, this study suggests that the well-established association found for prenatal maternal anxiety and child behavior difficulties was no longer present, once stable confounding factors was accounted for in a sibling comparison design. Our findings push forward our thinking about early experiences and precursors for child difficulties in the first five years of life. Pregnant women of today may feel more confident that fetal psychological experiences may be less risky than previously thought because we have improved the study design and methodology.

## Conclusions

In this large prospective cohort, we found that both maternal and paternal anxiety were associated with behavior problems, after adjusting for multiple controls. However, this association was attenuated within full-sibling pairs. Our findings suggest that the association between prenatal maternal anxiety and behavioral difficulties is confounded by genetic and/or other family factors.
